# Rumination syndrome in children and adolescents: a school survey assessing prevalence and symptomatology

**DOI:** 10.1186/1471-230X-12-163

**Published:** 2012-11-16

**Authors:** Shaman Rajindrajith, Niranga Manjuri Devanarayana, Bonaventure Jayasiri Crispus Perera

**Affiliations:** 1 Department of Paediatrics, Faculty of Medicine, University of Kelaniya, Thalagolla Road, Ragama 11010, Sri Lanka; 2 Department of Physiology, Faculty of Medicine, University of Kelaniya, Thalagolla Road, Ragama, 11010, Sri Lanka; 3 Postgraduate Institute of Medicine, University of Colombo, Colombo, Sri Lanka

**Keywords:** Adolescent, Child, Epidemiology, Functional gastrointestinal disorder, Rumination syndrome

## Abstract

**Background:**

Rumination syndrome (RS) is a functional gastrointestinal disorder (FGD) increasingly recognized in children and adolescents. The epidemiology of this condition in school aged children is poorly understood. The main objective of this study was to assess the prevalence of rumination and other related associations in a cohort of Sri Lankan children.

**Methods:**

Children aged 10-16 years were randomly selected from 8 schools in 4 provinces in Sri Lanka. RS was diagnosed using Rome III criteria. Data was collected using a self administered questionnaire distributed in an examination setting. It was translated into Sinhala, the native language and pretested before distribution.

**Results:**

A total of 2163 children were included in the study (55% boys, mean age 13.4 years, SD 1.8 years). Prevalence of RS was 5.1% (*n* = 110); boys 5.1% and girls 5.0%. When symptoms were analyzed, 73.6% reported re-swallowing of regurgitated food, while the rest spat it out. In 94.5% regurgitation occurred during the first hour after the meal. Only 8.2% had daily symptoms while 62.7% had symptoms weekly. Abdominal pain, bloating and weight loss were the commonest symptoms associated with RS (19.1%, 17.3% and 11.8% respectively). No significant association was observed between exposure to stressful events and rumination (*p* > 0.05). Twenty (18.2%) with RS fulfilled Rome III criteria for at least one other FGD. School absenteeism was seen in 11.8% of affected children.

**Conclusion:**

RS was reasonably common in this cohort of school-aged children and adolescents in Sri Lanka. However, symptoms were severe enough to affect schooling only in 12% of affected children. Around one fifth with RS had at least one other overlapping FGD.

## Background

Rumination syndrome is characterized by effortless, repetitive and painless regurgitation of partially digested food into the mouth soon after a meal, which is subsequently re-chewed and re-swallowed, or else spat out [1]. Typically, affected children do not get a feeling of nausea or retching and do not regurgitate during sleep. They respond poorly to standard treatment for gastro-oesophageal reflux.

Rumination syndrome is one of the less commonly recognized functional gastrointestinal disorders in the paediatric age group. The diagnostic criteria for rumination syndrome were first described for infants less than 8 months in Rome II criteria [2] and only in its latest version (Rome III), has the rumination syndrome been recognized in other age groups [1]. In clinical settings it is probably under diagnosed due to insufficient awareness among clinicians. Moreover, rumination is frequently misdiagnosed as gastro-oesophageal reflux disease, recurrent vomiting and upper gastrointestinal motility disorders such as gastroparesis. Poor understanding of clinical characteristics of this disorder leads to delay in diagnosis and treatment which often has far reaching consequences such as weight loss, malnutrition, dental erosions, halitosis and electrolyte disturbances, together with resulting significant functional disabilities [3]. A noteworthy percentage of affected children have other physical or psychological illnesses [4].

Rumination was believed to be common among children and adolescents with developmental abnormalities and learning difficulties [5,6]. However, it is now increasingly recognized in individuals with normal cognitive abilities [7,8]. Epidemiological data on this clinical entity is lacking and most of the available data are from case series from referral centres [3,4,7,8]. A preliminary study conducted in a semi-urban school in Sri Lanka has reported rumination syndrome in 4% of 12-16-year old children [9]. No other study has attempted to assess the prevalence and clinical characteristics of this important disorder in Sri Lankan children.

The main objectives of this study were to assess the prevalence of rumination in Sri Lankan children aged 10 to 16 years and to identify psychosocial and family related factors associated with this condition.

## Methods

### Study population

This was a prospective cross sectional survey conducted in Sri Lanka. Four provinces were randomly selected out of 9 provinces of the country. Two schools each, one urban and one rural, were selected from each province (total of 8 schools). From each school, 12 classes each were selected from academic years/grades 6 to 11 with two being randomly selected from each academic year. All the students in the selected classes, who were present on the day of the survey, were included in the study. School administration and parents were informed of the survey and consent was obtained before distribution of the questionnaire. Consent was also obtained from all children who participated in this study.

### Data collection

Data was collected using a self-administered questionnaire, distributed in an examination setting, to ensure confidentiality and privacy. It was distributed in the class room and collected immediately on completion. Research assistants were present while filling the questionnaire and verifications and help were provided to complete the questionnaire.

The questionnaire was in three parts. First part included questions on socio-demographic and family characteristics. Second consisted of questions on family and school related stressful life events the subjects were exposed to during the previous three months. This part of the questionnaire has been developed by Devanarayana et al. and validated for Sri Lankan children [9]. The third was the Rome III diagnostic questionnaire for pediatric functional gastrointestinal disorders - self-report form for children and adolescents (10 years of age and older) [10], translated into the native language (Sinhala). This questionnaire has been validated and used for Sri Lankan children of the same age group [11].

### Diagnosis, definitions and exclusion criteria

All the questionnaires were scrutinized by SR and NMD. Children who fulfilled Rome III criteria for rumination syndrome were identified using the guidelines given by the Rome III Committee.

Children were considered to have rumination syndrome when all of the following criteria were seen at least once a week for at least two months [1].

1. Repeated painless regurgitation and rechewing or expulsion of food that

a. begin soon after ingestion of a meal

b. do not occur during sleep

c. do not respond to standard treatment for gastroesophageal reflux

2. No retching

Children with a history of neurological problems, learning difficulties, autism, chronic neurodegenerative disorders and other chronic organic diseases which need long term medications were not included into the study.

### Data analysis

The data were analyzed using EpiInfo (EpiInfo 6, version 6.04 (1996), Centers for Disease Control and Prevention, Atlanta, Georgia, USA and World Health Organization, Geneva, Switzerland). Continuous and categorical data were described using means, standard deviations and percentages. Chi-square test was used to assess the association between sex, stressful life events, symptoms and rumination syndrome. *p* < 0.05 was considered as significant.

### Ethical approval

This study protocol was approved by the Ethical Review Committee, Sri Lanka College of Paediatricians.

## Results

A total of 2180 questionnaires were distributed and 2163 (99.2%) properly filled questionnaires were included in the analysis. Of them 1189 (55.0%) were males. Age ranged from 10 to 16 years, with a mean age of 13.4 years (SD 1.8 years). Seventeen incompletely filled questionnaires were removed from the analysis.

### Prevalence of rumination syndrome

One hundred and ten (5.1%) children fulfilled symptom based Rome III criteria for adolescent rumination syndrome. Prevalence of rumination was 5.1% (*n* = 61) in males and 5.0% (*n* = 49) in females. The difference was not significant (*p* = 0.91). Two thousand and fifty three children without rumination syndrome were considered as controls.

**Figure 1:**
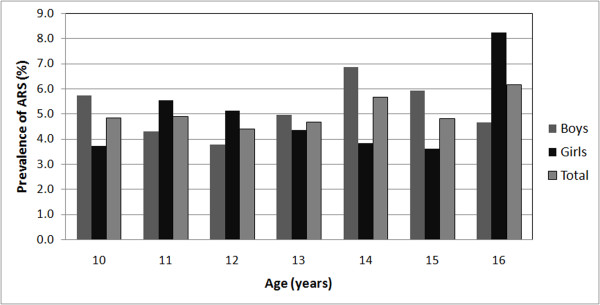
Prevalence of rumination syndrome according to age and sex.

Age specific prevalence of rumination is shown in Figure 1.

### Clinical characteristics of affected children

Out of 110 children with rumination, 81 (73.6%) reported re-swallowing of regurgitated food, while the rest [29 (26.4%)] spat it out. In 104 (94.5%) regurgitation had occurred during the first hour after ingestion of the meal. Sixty nine (62.7%) had symptoms of rumination at least once per week, 32 (29.1%) had symptoms several times per week, and 9 (8.2%) had daily symptoms.

Table 1 depicts the other symptoms associated with rumination syndrome. There was no significant difference in these symptoms between children with rumination and controls. Three patients reported nausea, but in none of them was it associated with regurgitation. None of the patients had vomiting or burning epigastric pain.

**Table 1:** Symptoms associated with rumination syndrome

**Symptom**	**Rumination syndrome (*n*** **= 110) Number (%)**	**Controls (*n*** **= 2053) Number (%)**
Abdominal pain	21 (19%)	252 (12.3%)
Abdominal bloating	19 (17.2%)	181 (8.8%)
Nausea	3 (2.7%)	133 (6.5%)
Loss of appetite	11 (10.0%)	159 (7.7%)
Loss of weight	13 (11.8%)	162 (7.9%)
Headache	10 (9.1%)	183 (8.9%)
Limb pain	9 (8.1%)	208 (10.1%)
Light headedness	6 (5.5%)	163 (7.9%)
Photophobia	2 (1.8%)	91 (4.4%)
Pallor	5 (4.5%)	51 (2.5%)

*p* > 0.05 for all comparisons between rumination syndrome and controls.

### Association between emotional stress and rumination

When the association between stressful life events and rumination was assessed, 72 (65.5%) children with rumination and 1316 (64.1%) controls were exposed to stressful life events during the previous 3 months. This was not significant (*p* = 0.85). Only stressful event significantly associated with this condition was a change in school [9 (8.2%) in children with rumination vs. 81 (3.9%) in controls, *p* = 0.03].

### Impact of rumination on daily activities

Thirteen (11.8%) had missed school and 15 (13.6%) had difficulty in sleeping. However, none had, sleeping disturbances due to symptoms of rumination.

### Other functional gastrointestinal disorders overlapping with rumination

Twenty (18.2%) children with rumination also fulfilled Rome III criteria for other functional gastrointestinal disorders. Eight of them (7.3%) had functional dyspepsia, 5 (4.5%) had irritable bowel syndrome, 5 (4.5%) had functional abdominal pain and 2 (1.8%) had functional constipation.

## Discussion

Due to lack of epidemiological studies, rumination syndrome has been previously considered as a rare paediatric disorder. In this study, for the first time we have described the epidemiology of rumination syndrome in a cohort of children and adolescents with normal intelligence. In this study, 5.1% of 10-16 year old children fulfilled Rome III criteria for adolescent rumination syndrome. There was no significant gender difference in prevalence of this condition and it was not significantly associated with emotional stress. Approximately 12% of children suffering from rumination syndrome had disturbances of daily activities. In 18%, rumination syndrome overlapped with other functional gastrointestinal diseases.

The first study that has assessed the prevalence of rumination syndrome was published in 1993 by Drossman et al. [12]. This is the first US householder survey using a questionnaire. In this study the prevalence among adults was noted to be 10.4%. Epidemiology of rumination syndrome in children has not been studied in detail. Most of the previous studies on rumination syndrome have been carried out in subjects with learning disabilities. It has been reported in 6 -10% of infants with developmental delay [13] and 8-10% of mentally handicapped adults [6,14]. These findings led to the belief that rumination is a condition predominantly seen in children and adults with learning difficulties. Subsequently this syndrome was increasingly recognized in children and adolescents with normal mental capabilities. A previous study assessing functional gastrointestinal disorders from our group has shown rumination syndrome in 4% of school aged children, using Rome III criteria [11], but the number of children with rumination syndrome in that study was not sufficient to describe clinical characteristics. In the present study we found rumination syndrome to be present in 5.1% of Sri Lankan children aged 10-16 years. This highlights the significant prevalence of rumination in children of normal intelligence.

The gender difference in prevalence of rumination syndrome has not been studied earlier. Even though, rumination has been reported in males [15,16], the majority of patients in previous hospital based studies and case reports on this disorder were females [3,17-19]. In a previous review of 147 children with rumination syndrome, 68% were females [4]. However, in the current study we did not observe a significant gender difference in the prevalence of rumination syndrome. Hospital based studies on functional gastrointestinal diseases are subjected to potential biases such as differences in healthcare seeking patterns. In fact females tend to seek healthcare more than that of males for functional gastrointestinal diseases such as irritable bowel syndrome [12] and that may be the possible reason for high percentage of females in previous hospital based studies and case reports.

Abdominal pain and weight loss are the commonest symptoms associated with rumination syndrome in children and adults [4,7,20]. We found similar results. However, percentage of children with abdominal pain, nausea and weight loss observed in our study is less than that reported by Chial *et al.* in their hospital based study (38% and 42.2% respectively) [4]. Furthermore, only 2 (2%) of our children with rumination syndrome had constipation compared to 21.1% seen in the previous study [4]. Associated symptoms such as pain, weight loss and alteration in bowel habits are troublesome symptoms and hence important determinants of health care consultation. Therefore, children with these symptoms are more likely to seek health care. This may be the reason for the high percentage of these symptoms observed in the previous hospital based study [4]. In contrast to this, our patients with rumination had higher percentage of bloating than reported in the previous study (4.1%).

Extraintestinal somatic symptoms such as headache and limb pain are known to be associated with functional gastrointestinal diseases in children [21,22]. In agreement with this, symptoms commonly seen in our children with rumination syndrome were headache, limb pain and light headedness. Presence of extraintestinal somatic symptoms is more suggestive of the presence of a functional disorder than an organic disorder such as gastro-oesophageal reflux. In addition, somatic symptoms can exaggerate associated disabilities and contribute to poor quality of life. Therefore, direct inquiry of these symptoms is important during evaluation of a child suspected of the rumination syndrome.

Emotional stress is known to modulate foregut motility such as gastric emptying in children with functional gastrointestinal diseases [23]. Emotional stress and other psychiatric disorders such as depression and anxiety are commonly reported in adult patients with rumination syndrome [24]. Therefore, we hypothesized that rumination syndrome is more common among children who were exposed to stressful life events. However, we failed to demonstrate a significant association between rumination syndrome and exposure to stressful life events. Only stressful life event which was associated with rumination syndrome was a change in school.

In a previous hospital based study, 72.7% of children with rumination syndrome have missed school due to symptoms [4]. However, school absenteeism is much less in our school based survey (11.8%). It is possible that children included in the hospital based study had more severe symptoms which led to school absenteeism.

Both children and adults with one functional gastrointestinal disease sometimes tend to have overlapping other functional gastrointestinal diseases at the same point in time [25,26]. The reason for this phenomenon is not entirely clear and it is possible that some FGD tend to share similar patho-physiological mechanisms such as abnormal visceral sensitivity, abnormal intestinal motility and malfunctioning brain-gut communications. In agreement with this, several other functional gastrointestinal disorders overlapped with rumination syndrome in our study and 18% of affected children fulfilled Rome III criteria for at least one other FGDs. Abdominal pain predominant FGD and constipation were the FGD overlapping with rumination syndrome in this study. In agreement with our study, a previous study using Rome III criteria and conducted in a secondary referral centre, showed rumination syndrome overlapping with irritable bowel syndrome, functional abdominal pain and functional constipation [27].

There are several strengths of our study. We have included a large number of children and adolescents in this study to get a valid cross-sectional cohort of paediatric population in Sri Lanka. We also had a significant number of children with rumination to describe their clinical profile in a meaningful way.

Rumination syndrome is an underappreciated condition in both adults and children [28,29]. This is due to misdiagnosis of rumination syndrome as having vomiting secondary to gastroparesis or gastro-esophageal reflux [28]. Lack of awareness among physicians may significantly contribute to under diagnosis [28,29]. Therefore it is not surprising that this has left a clinical impression that rumination syndrome is a rare disorder. However, our results show that a sizable proportion of children and adolescents have symptoms of rumination.

It has been stressed that eliciting key elements of the typical history is the most successful way to diagnose rumination syndrome [30,31]. Physiological studies such as 24 hour pH monitoring and impedance studies are helpful to identify the condition when clinical diagnosis is difficult [30]. It had been stated that gastroesophageal reflux, esophageal achalasia, gastroparesis, bulimia nervosa, and obstructive anatomical disorders must be excluded by appropriate diagnostic tests before making the final diagnosis of rumination syndrome [1]. Cardinal symptoms of reflux (painful regurgitation and burning epigastric pain) were not found in our sample of children with rumination. In addition organic conditions like achalasia and bulimia nervosa are rare diseases in children [32-34].

The main drawback of this study is that we did not carry out clinical evaluation and diagnostic tests to rule out these organic causes. It was practically impossible for us to carry out these investigations in a large epidemiological study involving over 2000 participants. In this backdrop, our results may over-represent the true prevalence of rumination syndrome in adolescents and caution is need when interpreting our results. Nonetheless, results of this study may help clinicians to be aware that symptoms of rumination are reasonably common in children and adolescents.

## Conclusions

This study highlights the significant prevalence of rumination syndrome in children and adolescents of normal intelligence. In this large school-based survey, we have shown rumination symptoms in 5.1% of children and adolescents aged 10-16 years in Sri Lanka. There is no significant gender difference in prevalence of rumination in children. However, the symptoms are severe enough to interrupt schooling only in 10% of affected children. Approximately one fifth of affected children suffer from other overlapping functional gastrointestinal disorders.

## Competing interests

The authors declare that they have no competing interests.

## Authors’ contribution

Dr SR and Dr NMD contributed equally for the study design, data collection, analysis, and preparation of the manuscript. Dr. BJCP contributed by critically analyzing the paper and providing significant intellectual contribution. All authors are in agreement with the contents of the manuscript.

## Pre-publication history

The pre-publication history for this paper can be accessed here:

http://www.biomedcentral.com/1471-230X/12/163/prepub
